# Expression and Functional Study of *BcWRKY1* in *Baphicacanthus cusia* (Nees) Bremek

**DOI:** 10.3389/fpls.2022.919071

**Published:** 2022-07-01

**Authors:** Meijuan Zeng, Yongjia Zhong, Zhiying Guo, Huiyong Yang, Haisheng Zhu, Liling Zheng, Yong Diao

**Affiliations:** ^1^School of Biomedical Sciences, Huaqiao University, Quanzhou, China; ^2^Crops Research Institute, Fujian Academy of Agricultural Sciences, Fuzhou, China; ^3^Root Biology Center, Fujian Agriculture and Forestry University, Fuzhou, China; ^4^Fujian Polytechnic Normal University, Fuqing, China; ^5^Department of Cardiovascular Surgery, First Hospital of Quanzhou Affiliated to Fujian Medical University, Quanzhou, China

**Keywords:** *Baphicacanthus cusia* (Nees) Bremek, *BcWRKY1*, bioinformatics analysis, metabolites, indole-related metabolism, flavonoid-related metabolism

## Abstract

*Baphicacanthus cusia* (Nees) Bremek (*B. cusia*) is an important medicinal plant. Its effective substances including indigo and indirubin are metabolites in indoleacetate metabolic pathway. Based on a previous transcriptome sequencing analysis, a WRKY transcription factor, BcWRKY1, in *B. cusia* was identified, showing significant correlation with effective substances from *B. cusia*. In this study, *BcWRKY1* was cloned by reverse transcription-polymerase chain reaction (RT-PCR). Further analysis showed that the *BcWRKY1* gene was 916 bp in length, containing three exons and two introns. The open reading frame (ORF) of *BcWRKY1* was 534 bp in length and encoded a WRKY domain-containing protein with 177 amino acids residues. Subcellular localization showed that BcWRKY1 protein was mainly localized in the nucleus. It could bind to the W-box motif and its role in transcriptional activation was confirmed in yeast. The function of BcWRKY1 was investigated by overexpressing *BcWRKY1* in *Arabidopsis thaliana*. Metabolic profiles in wild type and *BcWRKY1-OX1* transgenic *Arabidopsis thaliana* were analyzed with LC-MS. Results showed that the metabolic profile was significantly changed in *BcWRKY1-OX1 transgenic Arabidopsis thaliana* compared with wild type. Furthermore, indole-related metabolites were significantly increased in *BcWRKY1-OX1* transgenic *Arabidopsis thaliana*, and the metabolic pathway analysis showed that flavonoid biosynthesis was significantly enriched. Overexpression of *BcWRKY1* significantly changed flavonoid and indole metabolism and indole-related metabolites were significantly upregulated. We postulated that the BcWRKY1 transcription factor might be involved in the regulation of effective substances metabolism in *B. cusia*.

## Introduction

*Baphicacanthus cusia*(Nees) Bremek (*B. cusia*) (Supplementary Figure 1), also known as south isatis root, belongs to the *Acanthaceae* family (Huang et al., 2009). It has many medicinal properties (Lin et al., 2019). The leaves and stems of the plant are used to extract Indigo naturalis (Qing-Dai), while the roots are used for the production of traditional Chinese medicine (TCM), which has been recorded in the Chinese Pharmacopeia (China Pharmacopoeia Commission, 2020). Qing-Dai (indigo and indirubin) has the functions of purging fire, clearing heat, and detoxification, as well as analgesic and anti-inflammatory effects (Li et al., 2011). The roots of *B. cusia* and *Isatis indigotica Fortune* are termed as south and north isatis root, respectively, exhibiting antiviral, antibiosis and anti-inflammatory properties (Shen, 2009). Previous study has shown the effective substances in *B. cusia*. For example, indirubin can treat chronic myelogenous leukemia (Wang et al., 2014a), while its derivative 6-bromo indirubin-3′-oxime can effectively inhibit the growth of ovarian cancer cells (Yu and Zhao, 2015; Zeng and Diao, 2016). Other effective substances, such as indigo and tryptanthrin in *B. cusia*, were demonstrated to exhibit anti-inflammatory activities *in vitro* (Ishihara et al., 2000; Danz et al., 2001; Liu et al., 2014; Huang et al., 2017). Indigo and indirubin are belonging to indole alkaloids. It has been reported that the synthesis of indole alkaloids in plants is controlled by *DXR, SLS, G10H, TAA1, YUC1* and so on (Han et al., 2007; Wang et al., 2015). Flavonoids are isolated from the ethanol extract of *B*. cusia (Liu et al., 2009). Flavonoids are also important secondary metabolites with effective substance in medicinal plant, which is usually regulated by *PAL, CHS, CHI, ANR, FLS* and *FTH* (Qiao et al., 2009; Zou et al., 2016; Zhang et al., 2017). Taken together, *B. cusia* is an important TCM herb of Chinese traditional medicine and has been widely used to treat various diseases.

Transcription factors (TFs) are proteins that regulate gene expressions at the transcriptional level. It is reported that TFs participate in the regulation of essential physiological metabolism (Liu et al., 2001; Mitsuda and Ohme-Takagi, 2009; Ma et al., 2015). TFs regulate the expression of target genes through binding to cis-acting elements to activate or repress the expression of downstream genes (Priest et al., 2009). WRKY TF is one of the largest TF families in higher plants, which contain a conserved WRKYGQK sequence in the DNA-binding domain (Eulgem et al., 2000; Rushton et al., 2010; Wen et al., 2017b). It has been reported that WRKY TFs specifically bind to the cis-acting element W-box [(T)TGAC(C/T)] in the promoter region of the target gene through the WRKY domain, thus activating or inhibiting transcription and regulating the expression of downstream genes (Bakshi and Oelmüller, 2014; Huang et al., 2019). It is believed that WRKY TFs are widely involved in the responses to various biotic and abiotic stresses, and hormonal signaling, regulating plant growth and development (Xiong et al., 2014; Gu et al., 2015; Wani et al., 2016; Jiang et al., 2017; Finatto et al., 2018). Besides, more and more research has shown that WRKY TFs also participate in secondary metabolic regulation such as indole alkaloid production in plants (Xu et al., 2004; Suttipanta et al., 2011). Moreover, the preliminary results from our group indicated a significant correlation of *BcWRKY1* with indole metabolic pathway in *B. cusia* (Huang, 2017). Therefore, we postulated that *BcWRKY1* might be involved in the metabolism of effective substance in *B. cusia*. In this study, *BcWRKY1*, which showed a high association with indole metabolic pathway in *B. cusia*, was cloned, and its function was validated in plant *Arabidopsis* model, especially its influence on metabolite profiles and indole metabolic pathway in *Arabidopsis* was investigated. These results conferred the understanding of the role of *BcWRKY1* in regulating the effective substance in *B. cusia*.

## Materials and Methods

### Plant Material

The plant material, *B. cusia*, was collected from the Field experimental station of Huqiao University in Shufeng town of Fujian Province, China (25°25′N 118°39′E).

### Primer Design

Based on the sequencing data of *B. cusia* transcriptome (accession number SRR4428209) and the full length of *BcWRKY1* gene (c16427_g1_i1), the degenerate primers WRKY-F-XhoI and WRKY-R-BamHI were designed using Premier Premier 5.0 (Supplementary Table S2). The primer sequence was synthesized by Sunya Biotechnology Company (Fuzhou, China).

### Total RNA Extraction and cDNA Synthesis

Total RNA of *B. cusia* was extracted with TRIzol method according to the following protocol: A total of 0.1 g leaves of *B. cusia* were homogenized with liquid nitrogen. The homogenate was mixed with an additional 1 mL TRIzol and incubated at room temperature for 1 min. Subsequently, 200 μL chloroform was added to the mixture and incubated at room temperature for 3–5 min. The mixture was centrifuged at 12,000 ×*g* for 15 min at 4 °C; 500 μL of the supernatant was mixed with 500 μL isopropyl alcohol. Then 1 mL of 75% ethanol was added after discarding the supernatant and the mixture was centrifuged at 12, 000 ×*g* for 5 min at 4 °C, twice. The precipitate was air-dried, solubilized in 25–30 μL ddH_2_O, and maintained at 4 °C for 1–2 h (Ma et al., 2008). Finally, the RNA was analyzed by 1 % agarose gel electrophoresis and the quality and concentration were determined by Nanodrop 2000 UV-vis Spectrophotometer (Thermo Scientific, Wilmington, USA). The remaining was stored at −80 °C until utilized for cDNA synthesis by reverse transcription using Trans Script One-Step gDNA Removal and cDNA Synthesis Super Mix (Beijing TransGen Biotech Co., Ltd.) according to the manufacturer's instructions.

### PCR Amplification

The PCR reaction system included 25 μL 2 × Buffer, 5 μL dNTPs, 2 μL F1 primer, 2 μL R1 primer, 4 μL cDNA, and 1 μL enzyme, with 11 μL ddH_2_O in a reaction volume of 50 μL. The PCR reaction conditions were as follows: initial denaturation for 5 min at 95 °C, followed by 40 cycles of denaturation at 94 °C for 20 s, annealing at 58 °C for 20 s, and extension at 68 °C for 40 s. The final extension was carried out for 5 min at 68 °C. The PCR products were detected by 1 % agarose gel electrophoresis.

### Cloning and Sequencing of *BcWRKY1*

The target PCR fragment was recovered using Easy Pure Quick Gel Extraction Kit (Beijing TransGen Biotech Co., Ltd.). The DNA was eluted, and the recovered fragment was ligated to the pEASY-Blunt vector (Beijing TransGen Biotech Co., Ltd.) and transformed into Trans1-T1 (Beijing TransGen Biotech Co., Ltd.). The positive clones were sent to BoShang Biotechnology (Fuzhou, China) for sequencing.

### Bioinformatics Analysis

The ORF finder (NCBI) was used for identifying the ORF of *BcWRKY1* (Gao et al., 2009). Protparam (www.expasy.org/tools/protparam.html) was used for predicting the physical and chemical properties of the BcWRKY1 amino acid sequence (Wang et al., 2013). Eslpred (Bhasin and Raghava, 2004) (http://www.imtech.res.in/raghava/eslpred/) was used for predicting the subcellular localization of BcWRKY1 protein. DAS.TMfilter (https://mendel.imp.ac.at/DAS/) was used for analyzing the transmembrane protein structure (Cserzö et al., 2002). Signal P4.1 (http://www.cbs.dtu.dk/services/SignalP/) was applied to predict the signal peptide (Shi et al., 2012). Protein functional sites were analyzed by MotifScan (https://myhits.sib.swiss/cgi-bin/motif_scan). NPS@Network Protein Sequence Analysis (https://npsa-prabi.ibcp.fr/cgi-bin/npsa_automat.pl?page=/NPSA/npsa_seccons.html) was used to predict the secondary structure of the protein (Lv et al., 2016). SWISS-MODEL was used to predict the three-dimensional structure of BcWRKY1 (Guex and Peitsch, 1997; Schwede et al., 2003; Arnold et al., 2006). The search for protein sequence similarities of BcWRKY1 was conducted using BLAST algorithm at the National Center for Biotechnology Information (http://www.ncbi.nlm.nih.gov/blast). The neighbor-joining method (Mega 5.10) was used for constructing a phylogenetic tree (Shang et al., 2013; Kumar et al., 2016).

### Subcellular Localization

*BcWRKY1* was amplified from the cDNA of *B. cusia* with primers WRKY-for-5941GFP-F and WRKY-for-5941GFP-R (Table S2), cloned into the intermediate *5941-35S-GFP* vector. The *5941-35S-BcWRKY1-GFP* plasmid was constructed by inserting the *BcWRKY1* to the *Asc*I restrict sites of the *5941-35S-GFP* vector, and 5*941-35S-OsSPX1-RFP* was used a marker (Wang et al., 2014b). The positive clone was introduced into *Agrobacterium* EHA105, cultured, and injected into 4-week-old tobacco leaves, followed by culturing at 22–28 °C for 48 h. Then, the infected leaves were observed under a confocal laser scanning microscope.

### DNA Binding Assay

For the verification of DNA binding activity of BcWRKY1 as a WRKY family transcription factor, the Electrophoretic Mobility Shift Assay (EMAS) assay was employed. First, the *BcWRKY1* was sub cloned to the *pGEX-4T-1* prokaryotic expression vector using *EcoR*I restriction sites with primer pair GST-WRKY-F and GST-WRKY-R (Supplementary Table S2). and then the resulting vector *GST-WRKY* was transformed into the BL21 (DE3) cells. The expression and purification of recombinant protein was conducted according to the study by Lv et al. (2014). The classic WRKY binding motif W-box was synthesized and labeled with biotin by SUNYA company, and the sequence of ploynucleatides including W-box was listed in Supplementary Table S2. EMSA was performed using LightShift Chemiluminescent EMSA kit (Thermo Scientific) according to the manufacturer's instructions. The biotin-labeled probes were detected using chemiluminescence substrate (Thermo Scientific) and the ChemDoc XRS system (Bio-Rad).

### Transcriptional Activation Activity

The *BcWRKY1* gene was amplified by PCR using primers WRKY-R-for-BD-F and WRKY-F-FOR-BD-R, with 40 amplification cycles as previously described (Supplementary Table S2). The *pGBKT7-BcWRKY1* vector was constructed, and the *pGBKT7-BcWRKY1* and *pGBKT7* plasmids were respectively transformed into the AH109 competent cells, and then invertedly cultured at 30 °C for 48–72 h. Yeast plaques on SD/-trp medium were observed and photos were taken.

### Real-Time Fluorescence Quantitative PCR

Total RNA were extracted from wild type and transgenic *Arabidopsis* overexpressing *BcWRKY1* gene using RNAiso Plus reagent (TaKaRa Bio). The cDNA was further synthesized through reverse transcription reaction and quantitative PCR using *TransStart*® Tip Green qPCR SuperMix (TransGen Biotech, Beijing), according to the instruction of the manufacturer. Real-time fluorescence quantitative PCR was carried out on the LightCycler96 (Roche Diagnostics) PCR system. Additionally, qPCR reactions were carried out in 25 μL reaction system containing 10 μL SYBR Premix, 0.4 μL forward and reverse primers, 4 μL cDNA template, and 0.4 μL Rox, using ddH_2_O to adjust to 25 μL. The PCR program were set as 94 °C for 5 min, followed by 44 cycles of 94 °C 20 s, 58 °C 20 s, and 72 °C 20 s. Totally, there were three replicates for each biological analysis. The *Actin* gene was selected as a reference gene (Sun et al., 2007). The expression of BcWRKY1 was calculated using the methods described by Livak and Schmittgen (2001). Primers used for qPCR are listed in Supplementary Table S2.

### Gene Function Verification

*BcWRKY1* overexpression transgenic *Arabidopsis thaliana* line was obtained by transforming recombinant plasmid *5941-35S-BcWRKY1-GFP* into Rdr6 wild type *Arabidopsis thaliana* using *Agrobacterium*-mediated floral dipping method (Bent, 2006). The collected T1 generation seeds on MS medium containing Glufosinate were cultured in an artificial growth chamber and the positive transgenic seedlings were further identified with fluorescence microscopy (22 °C 18 h in the day, 20 °C 6 h at night). The metabolites in wild type and *BcWRKY1-OX1* transgenic *Arabidopsis thaliana* (T2 generation) were analyzed by UPLC-MS at the Novogene Institute (Beijing, China). The offline data (raw-data) file was imported into the CD (Compound Discoverer TM2.0) search software to simply screen the retention time and mass-charge ratio, followed by peak alignment of different samples using a retention time deviation of 0.2 min and a mass deviation of 5 ppm (Thevenot et al., 2015). Peak extraction was carried out according to a set quality deviation of 5 ppm, a signal strength deviation of 30 %, the signal noise ratio, the minimum signal strength (100, 000), adduct ion and other information (Fraga et al., 2010; Chen et al., 2013; Zhu et al., 2013). At the same time, peak area was quantified, target ions were integrated, molecular formula was predicted and compared with mzCloud database (Ruttkies et al., 2016), background ions were removed by blank samples, quantitative results were normalized by QC samples, and finally the data identification and quantitative results were obtained. The experimental samples and QC samples were extracted respectively, and the corresponding peaks were obtained. After Pareto-scaling treatment, the data were analyzed by PCA. Partial Least Squares Discrimination Analysis (PLS-DA) was used to establish the PLS-DA model for each group (Chen et al., 2009; Tang et al., 2014). The Variable Importance in the Projection (VIP) value of the first principal component of PLS-DA model was used, and the *P* value obtained by *T*-test analysis was used to screen the differential metabolites between the two experimental groups (Thevenot et al., 2015), and the differential metabolites analysis table was made. A volcano map was plotted according to log_2_ (fold change) and -log_10_ (*p*-value). Considering KEGG Pathway as a unit, hypergeometric test was used to find out significantly enriched pathways associated with differential metabolites. The most important biochemical metabolic pathways and signal transduction pathways involved in differential metabolites could be determined by Pathway significant enrichment, and the KEGG pathway enrichment analysis map was drawn (Kanehisa and Goto, 2000; Wen et al., 2017a).

### Statistical Analysis

Means and SE (Standard Error) values were calculated using GraphPad Prism version 7.0 (GraphPad Software Inc., San Diego, CA, USA; https://www.graphpad.com). The significantly differential metabolites between two groups were analyzed with DEseq2 package in R with absolute log_2_Foldchange > 1. Adjusted *P* value was calculated with FDR (False discovery rate). The two-tailed student's *t* test was used to compare the two samples.

## Results

### Cloning and Characterization of *BcWRKY1*

For the cloning of the *BcWRKY1*, the primers specifically for *BcWRKY1* were designed according to the sequence of *BcWRKY1* obtained through RNA-seq (Huang, 2017). PCR was performed with cDNA of *B. cusia* leaves using specific primer pair designed. To investigate the structure of *BcWRKY1*, PCR was also performed with genomic DNA of *B. cusia*. The results showed that the length of *BcWRKY*1 gene was 916 bp, containing a 534 bp ORF (Supplementary Figure 2A). *BcWRKY1* contained three exons, size 257 bp, 151 bp, and 126 bp, respectively and two introns, 154 bp and 228 bp, respectively (Supplementary Figure 2B). *BcWRKY1* gene encoded a protein with 177 amino acids residues (Supplementary Figure 2C). The composition and physicochemical characteristics of the deduced BcWRKY1 protein are compared in Supplementary Table S1.

Eslpred software prediction showed that BcWRKY1 protein was localized in the nucleus. DAS.TMfilter was further used to predict the transmembrane structure of the BcWRKY1 protein. The results showed that this protein did not have any transmembrane domains. Signal P4.1 prediction showed that BcWRKY1 did not contain a signal peptide. Motif Scan analysis showed that the protein contained a conserved WRKY domain (115-174 bits, E-value 2.6e-38). Network Protein Sequence Analysis (NPS) was used to predict the secondary structure of the protein, and the results demonstrated the presence of alpha helix (20.79 %), extended strand (15.73 %), and random coil (62.92 %) components (Supplementary Figure 3). These findings indicated that the secondary structure of BcWRKY1 primarily consisted of random coil and alpha-helix. The three-dimensional structure of BcWRKY1 obtained by SWISS-MODEL is shown in Supplementary Figure 2D.

### Phylogenetic Analysis

Genes with close homology to BcWRKY1 were searched in NCBI and their protein sequences were downloaded to construct a phylogenetic tree with MEGA 5.10 software. ClustalX was used for multiple alignments and the Neighbor-Joining method with 1000 replicates of bootstrap testing (Saitou and Nei, 1987; Shang et al., 2013). The phylogenetic tree of BcWRKY1 showed that the BcWRKY1 of *B. cusia* was closely related to WRKY in *Helianthus annuus* (Supplementary Figure 2E). The results of multiple sequence alignment showed that the BcWRKY1 protein was highly conserved compared with its homologous proteins in *Helianthus annuus, Ziziphus jujuba, Olea europaea var. Sylvestris, Prunus persica* and *Phoenix dactylifera*, with a homology of 72.62, 73.17, 71.91, 73.68, and 75.34%, respectively (Supplementary Figure 4).

### Subcellular Localization of the BcWRKY1 Protein

**Figure 1:**
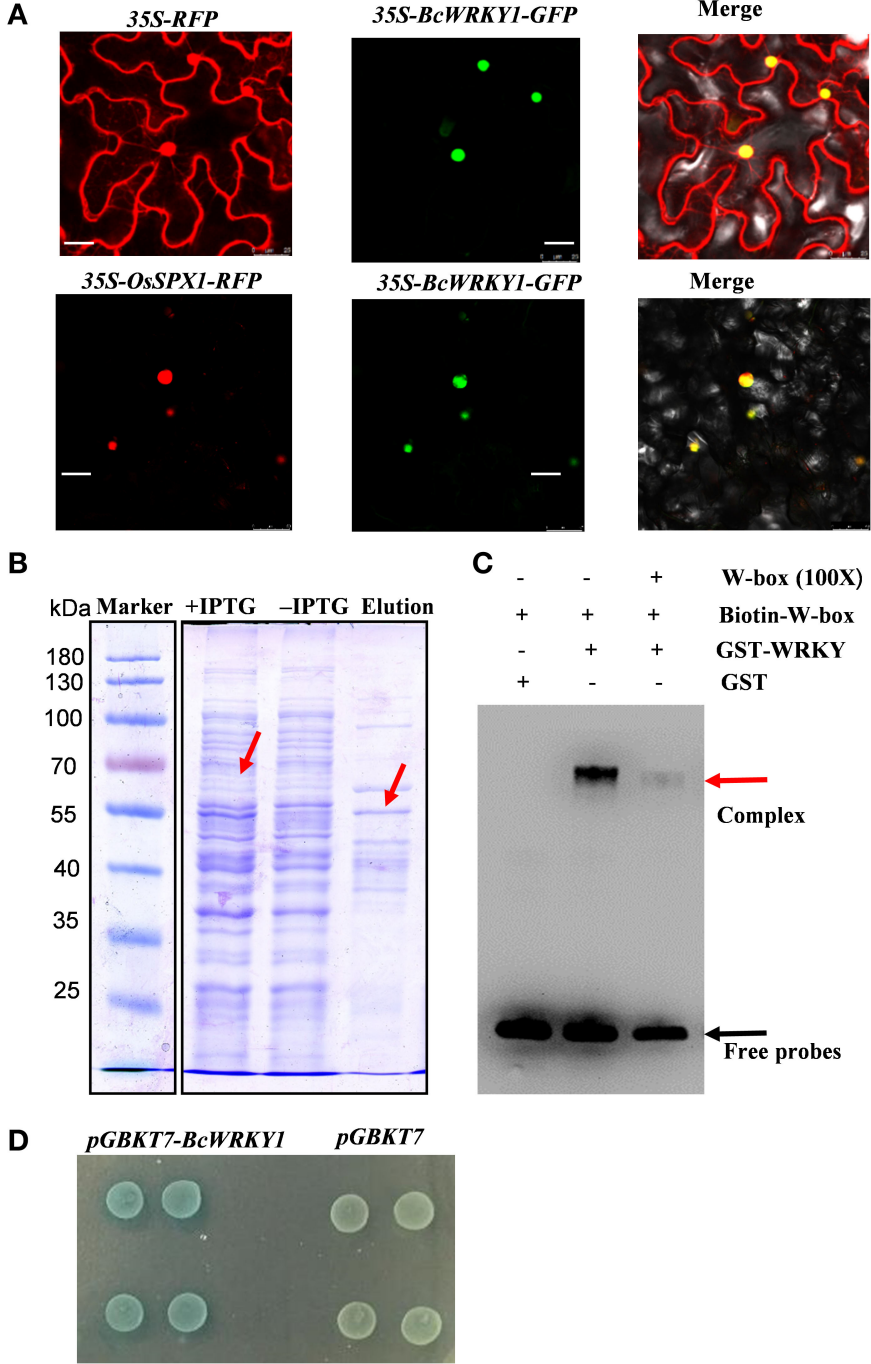
Expression and functional characterization of BcWRKY1 transcription factor. **(A)** Subcellular localization of 35S-BcWRKY1-GFP and 35S-RFP and subcellular co-localization of 35S-BcWRKY1-GFP and 35S-OsSPX1-RFP. **(B)** The results of recombinant protein GST-WRKY1 expression and purification. The molecular weight of the recombinant protein is indicated using protein markers. **(C)** The result of DNA binding activity of GST-WRKY1 to the DNA probe including W-box using EMSA assay. **(D)** Transcriptional activation activity of BcWRKY1 in yeast.

The vector containing *5941-35S-BcWRKY1-GFP* and the empty vector containing *5941-35S-RFP* were transformed into tobacco leaves for transient expressions of *5941-35S-BcWRKY1-GFP* and *5941-35S-RFP*. Then the tobacco leaves were cultured for 48 h, and observed under a confocal laser scanning microscope with GFP and RFP double channels. The results showed that *5941-35S-RFP* was localized in both the nucleus and cytoplasm of tobacco epidermal cells (bright points), and *5941-35S-BcWRKY1-GFP* was localized in the RFP bright spot where the nucleus was labeled (Figure 1A). This finding was in line with the protein subcellular localization prediction results with Eslpred, which suggested that BcWRKY1 protein was most likely to be in the nucleus. Meanwhile, *BcWRKY1-GFP* was further co-labeled with *5941-35S-OsSPX1-RFP* that was reported to be specially localized in the nucleus (Wang et al., 2014b). In addition, *OsSPX1-RFP* was detected in the nucleus, perfectly overlapping with the signal of *BcWRKY1-GFP* (Figure 1A). Taken together, these results demonstrated that BcWRKY1 protein was localized in the nucleus.

### DNA Binding and Transcriptional Activation Activity of BcWRKY1

Since BcWRKY1 was a putative WRKY TF, it was further investigated whether BcWRKY1 could bind to the DNA sequence including classic W-box motif. Recombinant protein GST-BcWRKY1 was expressed and purified in *E. coil* BL21 (DE3). The results showed that GST-BcWRKY1 expression was significantly induced by the addition of IPTG (Isopropyl-β-D-thiogalactoside), and the GST-BcWRKY1 recombinant protein was successfully purified (Figure 1B). Further analysis with EMSA showed that GST-BcWRKY1 could specially bind to the DNA probe including the W-box motif, and the binding of GST-BcWRKY1 to the biotin-labeled probe could be specially attenuated by the addition of unlabeled competitor DNA probe (Figure 1C). Furthermore, to investigate whether BcWRKY1 is a transcriptional repressor or an activator. Plasmids containing *pGBKT7-BcWRKY1* and *pGBKT7* were respectively transformed into yeast AH109 competent cells, and then cultured at 30 °C for 48–72 h. The plaque of AH109 (*pGBKT7-BcWRKY1*) and AH109 (*pGBKT7*) were similar in size on SD/-trp medium. The results showed that pGBKT7-BcWRKY1 fusion protein had no toxicity to AH109 cells. Besides, yeast AH109 (*pGBKT7-BcWRKY1*) showed blue plaque (Figure 1D), suggesting that BcWRKY1 protein could promote gene transcriptional activities.

### Overexpression of BcWRKY1 in *Arabidopsis thaliana*

**Figure 2:**
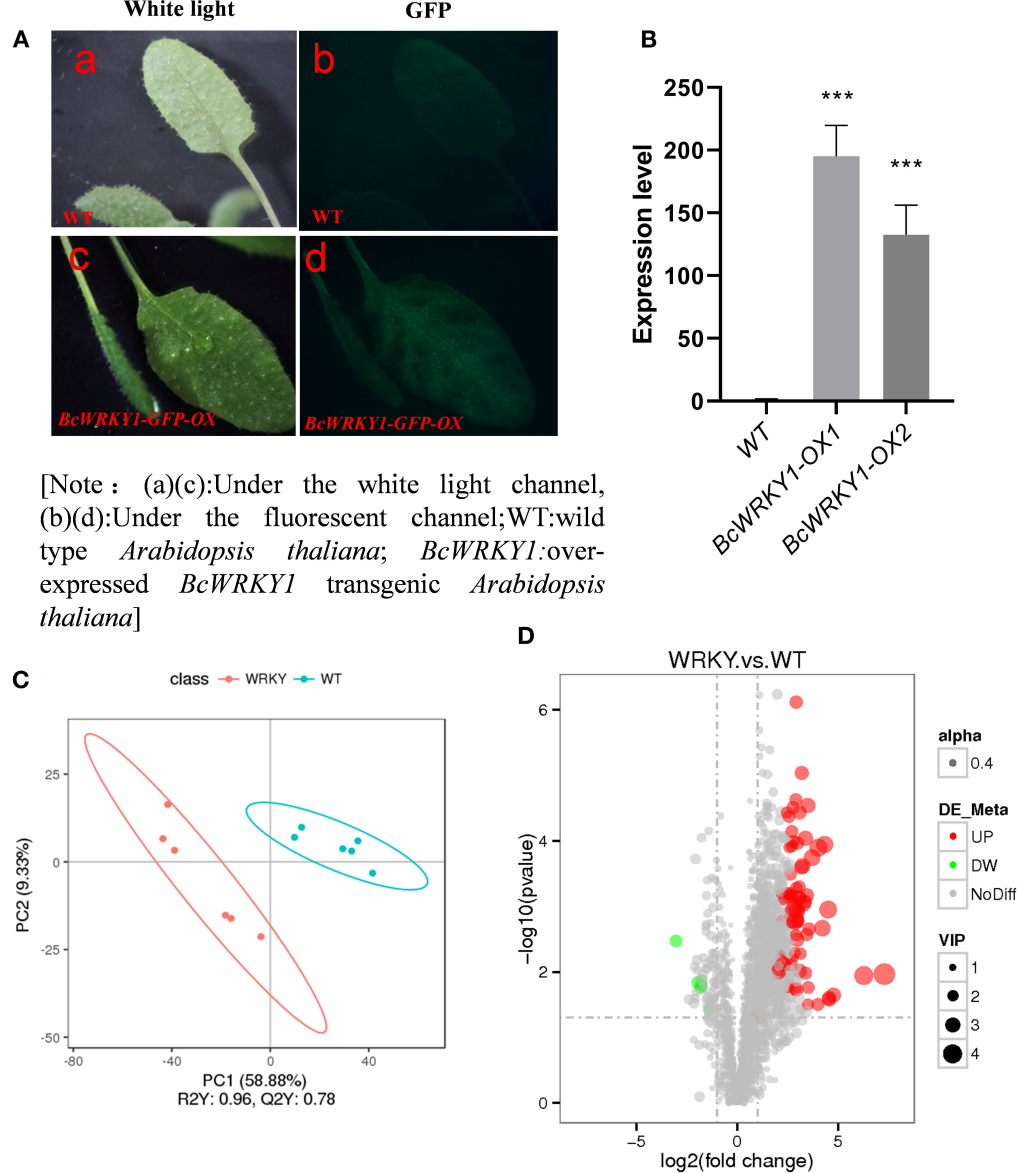
BcWRKY1 overexpression influences the metabolic profile in *Arabidopsis thaliana*. **(A)** Phenotypic overexpression of BcWRKY1-GFP in *Arabidopsis thaliana*; [Note: (a, c) Under the white light channel, (b, d) Under the fluorescent channel; WT: wild type *Arabidopsis thaliana*; BcWRKY1: BcWRKY1 overexpression transgenic *Arabidopsis thaliana*]. **(B)** The quantitation of expression levels of BcWRKY1 in transgenic *Arabidopsis thaliana* using qRT-PCR. **(C)** PLS-DA of differential metabolites between wild type and *BcWRKY1-OX1* transgenic *Arabidopsis thaliana* in positive ion mode; **(D)** Volcano Plots of differential metabolites between wild type and *BcWRKY1-OX1* transgenic *Arabidopsis thaliana* in positive ion mode, drawn by DEseq2 R package using a absolute log_2_Foldchange > 1 and an adjusted *P* value < 0.05 (FDR).

To investigate the function of BcWRKY1 protein, *BcWRKY1* was over-expressed in *Arabidopsis thaliana*. Positive transgenic *Arabidopsis* plants that grew normally on MS medium containing Glufosinate were then transplanted into organic soil spiked with vermiculite, and cultured in a growth chamber for a period of time. The plants with resistance to Glufosinate were sampled and observed under a fluorescence microscope. The positive transgenic plants were further identified by GFP signal detection in the leaves (Figure 2A). Two independent transgenic lines showing strong GFP signal were further selected to determine the expression levels of *BcWRKY1*. The results showed that the expression of *BcWRKY1* was significantly higher than that in the control line (Figure 2B). Hence, the transgenic *Arabidopsis* plant overexpressing *BcWRKY1-OX1* with strong GFP signal was selected for the following research.

### Differential Metabolic Profile Between Wild Type and *BcWRKY1-OX1* Transgenic *Arabidopsis thaliana*

To investigate the function of *BcWRKY1* in *Arabidopsis thaliana* metabolism, the metabolic profile in *Arabidopsis thaliana* was determined and compared between the wild type (WT) and *BcWRKY1-OX1* transgenic lines. The results from the Principal Component Analysis (PCA) showed that the metabolites in wild type and *BcWRKY1-OX1* transgenic *Arabidopsis thaliana* could be clearly separated by PC1 with an explanatory degree of 58.88%, followed by PC2, with an explanatory degree of 9.33% (Figure 2C). These results suggested that overexpression of *BcWRKY1* significantly changed the metabolic profile in *Arabidopsis thaliana*.

### Metabolites Regulated by the Expression of *BcWRKY1*

The Variable Importance in Projection (VIP) *P*-value of the first principal component in the PLS-DA model was used to identify differential metabolites by *t*-test. The threshold was set as VIP > 2.0, and the difference multiple FC was set to be > 2.0 or < 0.5. A *P*-value < 0.05 was used to identify the differential metabolites in wild type and *BcWRKY1-OX1* transgenic *Arabidopsis thaliana*.

The total number of identified metabolic compounds in both lines was 2,131. In Volcano Plots, gray points represented the metabolites without significant differences (NoDiff), and the total number of significantly different metabolites between wild type and *BcWRKY1-OX1* transgenic *Arabidopsis thaliana* was 80 (Figure 2D and Supplementary Table S3). Red points represented up-regulated metabolites in *BcWRKY1-OX1* transgenic line (UP), and the number of significantly up-regulated metabolites was 74. Green points represented down-regulated metabolites in *BcWRKY1-OX1* transgenic line (DW), and the number of significantly down-regulated metabolites was 6. The size of the dot represented the VIP value, and alpha (0.4) represented the transparency of points. Compared with wild type *Arabidopsis thaliana*, the number of up-regulated metabolites in *BcWRKY1-OX1* transgenic *Arabidopsis thaliana* was significantly higher than that of down-regulated metabolites (Figure 2D). In addition, significantly altered metabolites in WT and *BcWRKY1-OX1* transgenic lines were further analyzed and clustered, and the results showed that the differential metabolites between the two plant lines varied (Supplementary Figure 5). Furthermore, some differential metabolites exhibited significant correlation with each other (Supplementary Figure 6).

### KEGG Pathway Enrichment Analysis in Wild Type and *BcWRKY1-OX1* Transgenic *Arabidopsis thaliana*

In order to investigate which metabolic pathway these differential metabolites were enriched in, KEGG analysis was performed. It was clear that the differential metabolites in wild type and *BcWRKY1-OX1* transgenic lines participated in the main biochemical metabolic pathways and signal transduction pathways. KEGG Pathway enrichment analysis in wild type and *BcWRKY1-OX1* transgenic *Arabidopsis thaliana* are shown in Supplementary Table S4.

**Figure 3:**
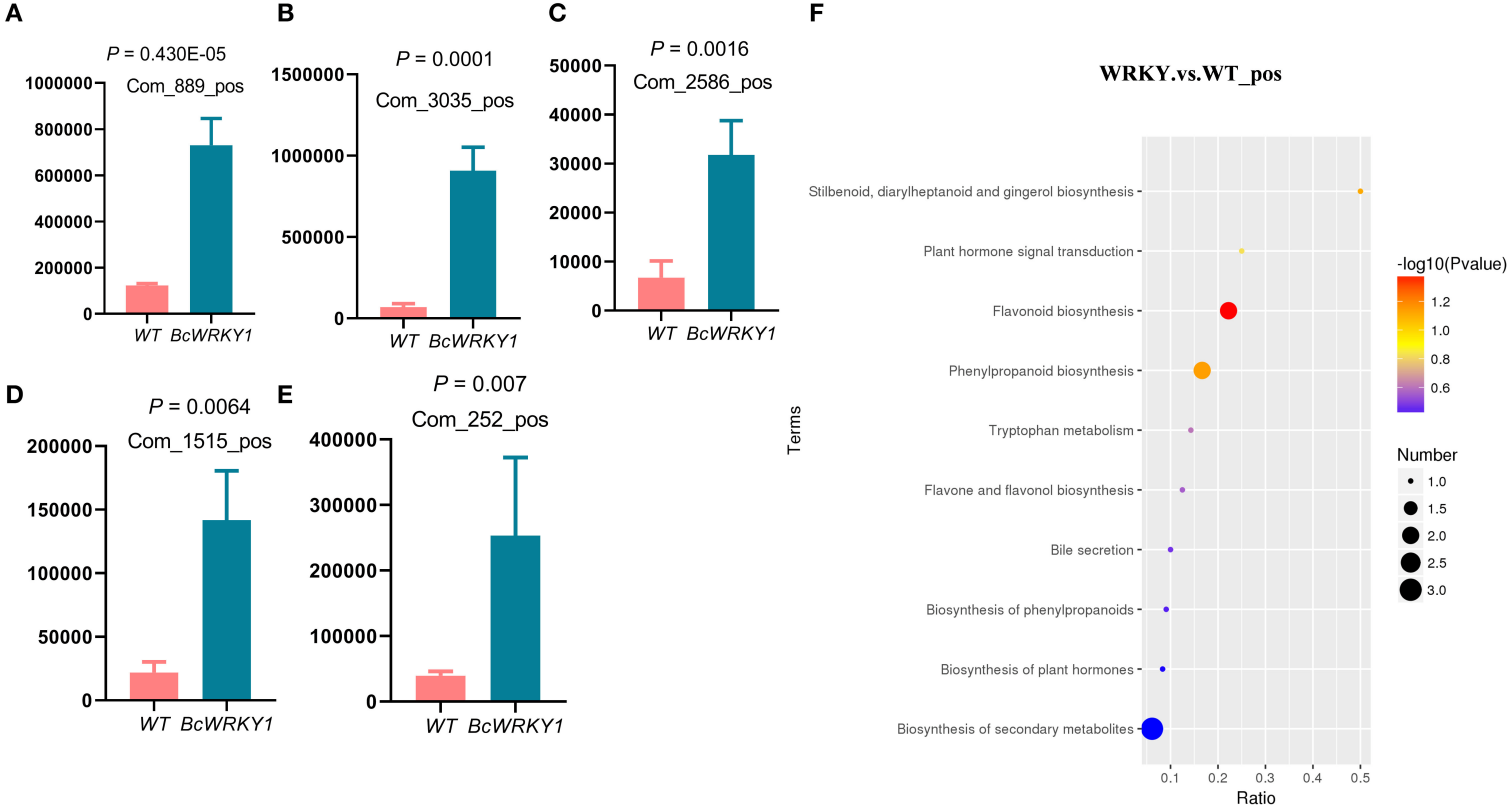
Indole-related metabolites are significantly increased in *BcWRKY1-OX1* transgenic *Arabidopsis thaliana* line. **(A**–**E)** Abundance of indole-related metabolites in wild type and *BcWRKY1-OX1* transgenic *Arabidopsis thaliana* line. The *P* value < 0.05 indicates a significant difference between the two groups determined using student's *t* test. **(F)** KEGG pathway enrichment analysis in wild type and *BcWRKY1-OX1* transgenic *Arabidopsis thaliana* in positive ion mode.

**Figure 4:**
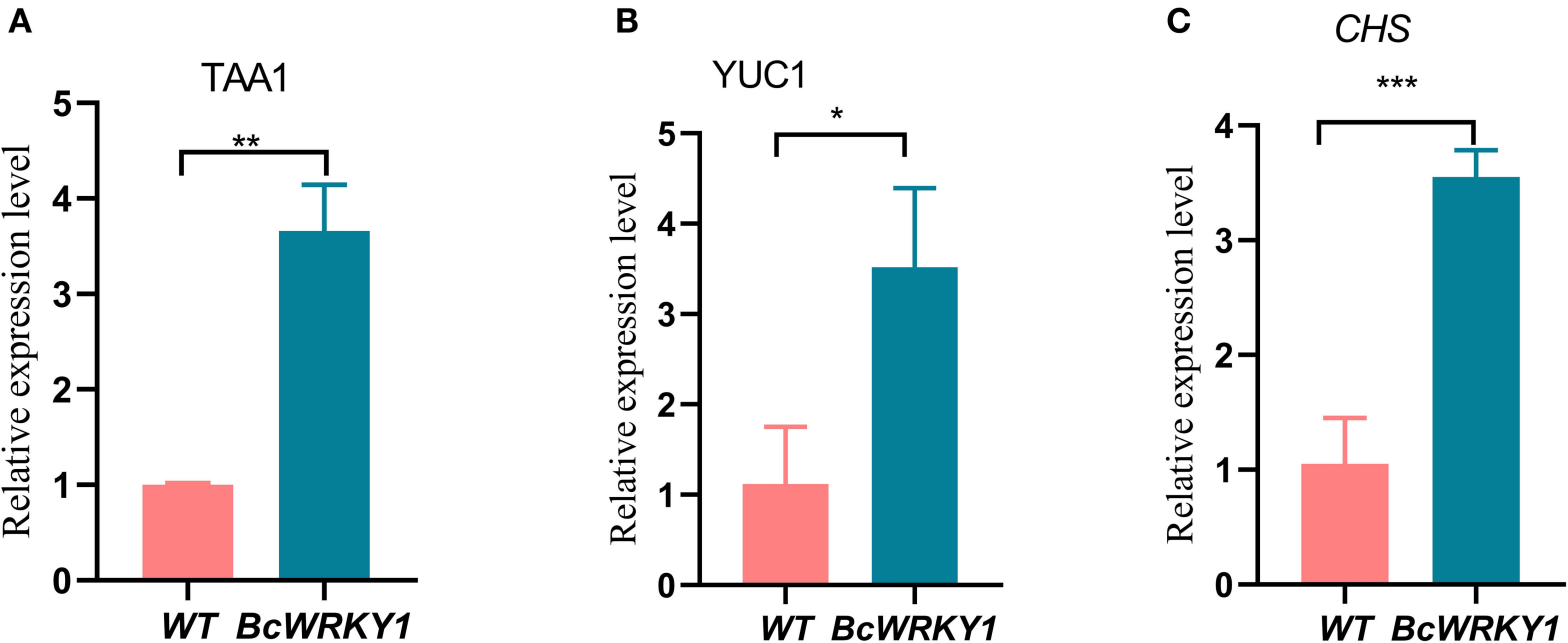
Expression analysis of key genes in the flavonoid and indole acetic acid biosynthesis pathway. **(A)** Expression analysis of *TAA1* in the biosynthesis of indole acetic acid pathway by qRT-PCR. **(B)** Expression analysis of *YUC1* in the biosynthesis of indole acetic acid pathway by qRT-PCR. **(C)** Expression analysis of *CHS* in the biosynthesis of flavonoid pathway by qRT-PCR.

The results showed that the abundance of indole-related metabolites were significantly increased in *BcWRKY1-OX1* line (Figures 3A–E) (Supplementary Table S5). Besides, there were more differential metabolites enriched in flavonoid biosynthesis and phenylpropanoid biosynthesis pathways in *BcWRKY1-OX1* transgenic line (Figure 3F). Compared with wild type *Arabidopsis thaliana*, the most obvious difference in *BcWRKY1-OX1* transgenic line was the enrichment of flavonoid biosynthesis (Figure 4F). Compared with wild type *Arabidopsis thaliana*, baimaside of flavone and flavonol biosynthesis in *BcWRKY1-OX1* transgenic *Arabidopsis thaliana* were significantly up-regulated (Supplementary Figure 7). Although tryptophan metabolism did not exhibit a significant change, the abundance of indoleacetate in the tryptophan metabolic pathway in *BcWRKY1-OX1* transgenic *Arabidopsis thaliana* was significantly increased (Supplementary Figure 8).

To further understand why *BcWRKY1* overexpression changed flavonoid and indole relative metabolites in *Arabidopsis thaliana*, we further detected the expression levels of key genes in the flavonoid and indole acetic acid biosynthesis pathways. Expression levels of key genes in the flavonoid and indole acetic acid biosynthesis pathway (such as *TAA1, YUC1* and *CHS*) were detected with qRT-PCR. Results showed that the expression level of *TAA1* and *YUC1* in indole acetic acid biosynthesis pathways in *BcWRKY1* overexpression line were significantly increased by comparison with wild type *Arabidopsis*. Similarly, expression of *CHS* in flavonoid biosynthesis in *BcWRKY1* overexpression line was also significantly increased (Figure 4). Taken together, our results suggested that BcWRKY1 might regulated the flavonoid and indole metabolism, through regulation the expression of key genes in these two biosynthesis pathway.

## Discussion

The development of molecular biological techniques has opened the avenue of the in-depth study of genes, which not only focuses on the gene function but also on mapping the genes in various plant systemic networks (Li and Zhou, 2014). Along with genome sequencing of different species, the *WRKY* gene family has also been identified in several species. WRKY is a kind of transcription factor with specific roles in plants (Cheng et al., 2012; Chi et al., 2013). Besides, our preliminary study suggested that the WRKY transcription factor exhibited a significant correlation with indole metabolic pathway in *B. cusia* (Huang, 2017). And the main effective substances including indirubin and indigo in *B. cusia* were the secondary metabolites derived from indole metabolism in *B. cusia* (Huang, 2017). More and more research has shown that WRKY TFs also participate in the secondary metabolic regulation such as indole alkaloid production in plants (Xu et al., 2004; Suttipanta et al., 2011). Therefore, based on the previous studies and the transcriptome sequencing database of *B. cusia*, the full length sequence of *WRKY* in *B. cusia* (*BcWRKY1*) was successfully cloned using RT-PCR, and bioinformatics analysis was performed (Supplementary Figure 2). Compared to the conventional laboratory-based experimental research, bioinformatics can obtain more reliable results in less time and was cost-effective. In subcellular localization studies, we found that BcWRKY1 protein was mainly localized in the nucleus, which was consistent with its function as a transcription factor. This result was also in line with the previous Eslpred prediction. The transcriptional regulation domain determined whether it could activate or inhibit gene expression (Dong et al., 2018). In the study, we found that BcWRKY1 protein could bind to the WRKY-binding motif W-box, acting as a transcriptional activator (Figures 1B–D), which was consistent with previous studies (Rushton et al., 1996). However, some WRKY proteins also play a role as transcriptional suppressor. For example, Bo (2016) found that tomato *SIDRW1* gene encoding the WRKY transcription factor might function as a transcriptional suppressor. In addition, the WRKY protein in some species, such as AtWRKY6, could play a dual role as transcriptional activator or repressor depending on the regulation process (Xie et al., 2005). Metabolomics is an effective method to comprehensively explore the distribution of compounds in plants, including differential metabolites (Tikunov et al., 2005). Target analysis method has high accuracy, but useful information may be missed in metabolic phenotype analysis (Wang et al., 2018). Non-targeted metabolomics has been applied to investigate the differential metabolites and analyze the enriched pathways, especially in studies on the discovery of metabolic markers. Due to high throughput characteristics and a wide coverage of metabolites (Duan et al., 2016), all cellular metabolites can be detected without bias, more comprehensively reflecting the overall metabolic state of cells (Patti et al., 2012). Non-targeted metabolomics LC-MS was used to analyze the metabolic profiles in wild type and *BcWRKY1-OX1* transgenic *Arabidopsis thaliana*. The results showed that flavonoid biosynthesis pathway was significantly influenced by *BcWRKY1* overexpression (Figure 3F). Besides, although tryptophan metabolism did not show a significant change (Figure 3F), some indole-related metabolites were significantly increased in *BcWRKY1-OX1* transgenic line (Figures 3A–E). These results suggested that *BcWRKY1* was involved in the regulation of indole metabolic pathways in *Arabidopsis thaliana* plant model. In addition, qRT-PCR suggested that indeed some genes in the flavonoid and indole biosynthesis pathway were induced in the *BcWRKY1-OX1* over-expression line. And indeed, the most homologous *WRKY* in *Arabidopsis thaliana* is *AtWRKY50*, which has been proved in regulation of sinapic metabolism in *Arabidopsis thaliana* (Hussain et al., 2018). However, whether *BcWRKY1* directly or indirectly regulated these processes needs further investigation. Besides, our findings indicated that *BcWRKY1* might regulate the corresponding metabolism pathways in *B. cusia*. Since the main effective substances in *B. cusia* were indigo and indirubin, both are products of indoles metabolism in the Tryptophan metabolismin *B. cusia* (Huang, 2017). Taken together, BcWRKY1 might participate in effective substances metabolism in *B. cusia*. These findings may lay a good foundation for further research about the effect of BcWRKY1 transcription factor on *B. cusia* metabolism.

## Conclusion

Effective substances metabolic pathway in *B. cusia* is still unknown. We identified a *BcWRKY1* involved in this pathway. We combined the use of bioinformatics analysis, transgenic plant model and metabolomics technology to study the function of BcWRKY1 protein in plant metabolism regulation. Overexpression of BcWRKY1 significantly changed flavonoid and indole-related metabolism and indole-related metabolites were also significantly upregulated. We postulated that BcWRKY1 transcription factor might be involved in the regulation of effective substances metabolism in *B. cusia*.

## Data Availability Statement

The raw data presented in the study are deposited in the MetaboLights repository, accession number (MTBLS4819).

## Author Contributions

MZ and YD conceived and designed the study. MZ performed the experiments and wrote the manuscript. MZ and YZ performed data analysis. MZ, ZG, HY, HZ, and LZ revised the paper. All authors contributed to the article and approved the submitted version.

## Funding

This work was supported by the Natural Science Foundation of China (Association of Science and Technology Cooperation Across the Taiwan Straits, Grant No. U1405215), Natural Science Foundation of China (Grant No. 82003903), Science and Technology Planning Project of Quanzhou (Grant No. 2019N031), and Science and Technology Planning Project of Quanzhou (Grant No. 2020C061). The role of the funding body in the design of the study, the collection, analysis and interpretation of data, and in writing the manuscript is managed and supervised.

## Conflict of Interest

The authors declare that the research was conducted in the absence of any commercial or financial relationships that could be construed as a potential conflict of interest.

## Publisher's Note

All claims expressed in this article are solely those of the authors and do not necessarily represent those of their affiliated organizations, or those of the publisher, the editors and the reviewers. Any product that may be evaluated in this article, or claim that may be made by its manufacturer, is not guaranteed or endorsed by the publisher.
