# Supplementary material for: Expression and Functional Study of BcWRKY1 in Baphicacanthus cusia (Nees) Bremek
Source: Front Plant Sci. 2022 Jul 1;13:919071. doi: 10.3389/fpls.2022.919071 (PMC9284225; doi:10.3389/fpls.2022.919071)
Supplement: Supplementary file 1 [file Image_1.pdf]

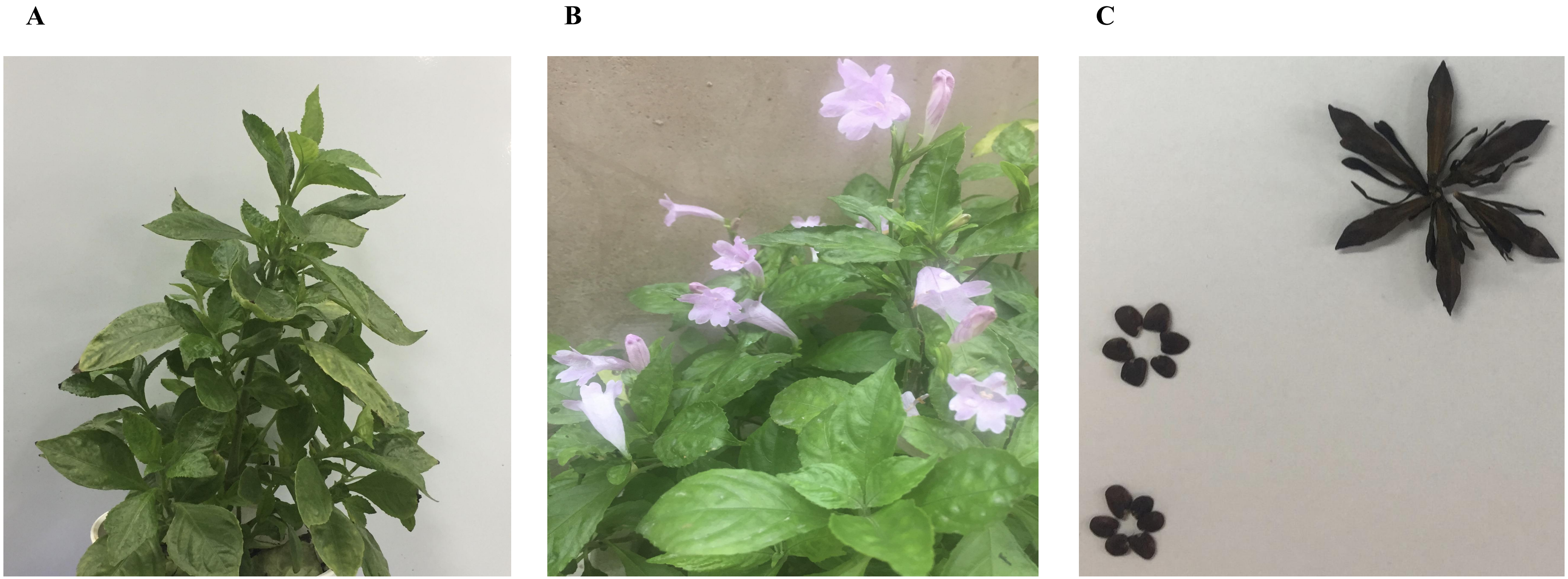

Supplementary Figure 1 Photograph of *B. cusia*.

(A) Leaves of *B. cusia*. (B) Flowers of *B. cusia*. (C) Pods and seeds of *B. cusia*.

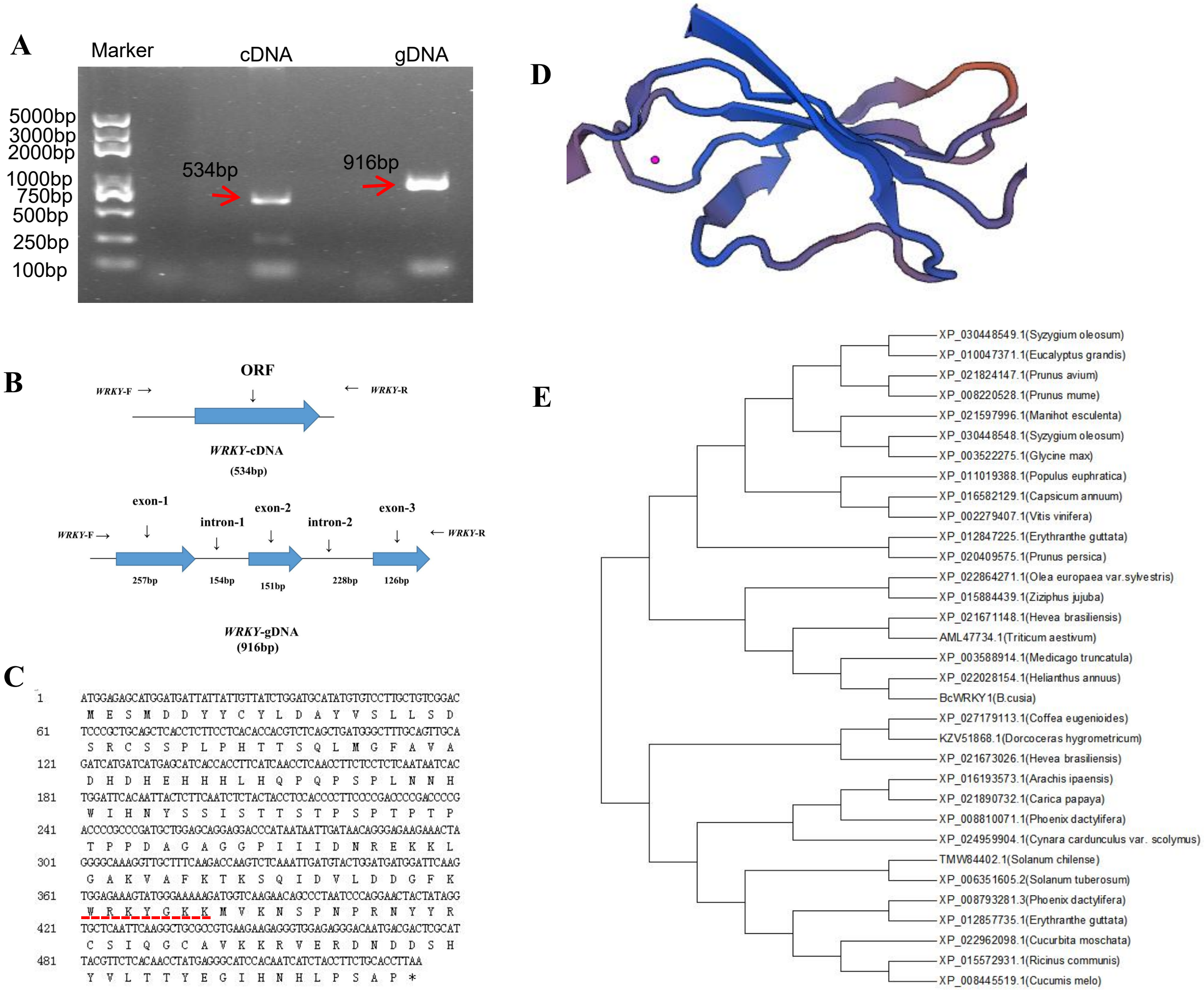

Supplementary Figure 2 Cloning and characterization of BcWRKY1.

(A)Cloning BcWRKY1 from cDNA and genome DNA of B. cusia. 5K DNA markers are used to indicate the size of the PCR production amplified from cDNA and DNA; (B) Gene structure analysis of BcWRKY1; (C) Deduced amino acid sequence of BcWRKY1 from B. cusia; (D) The three-dimensional structure of BcWRKY1; (E) Phylogenetic analysis of BcWRKY1.

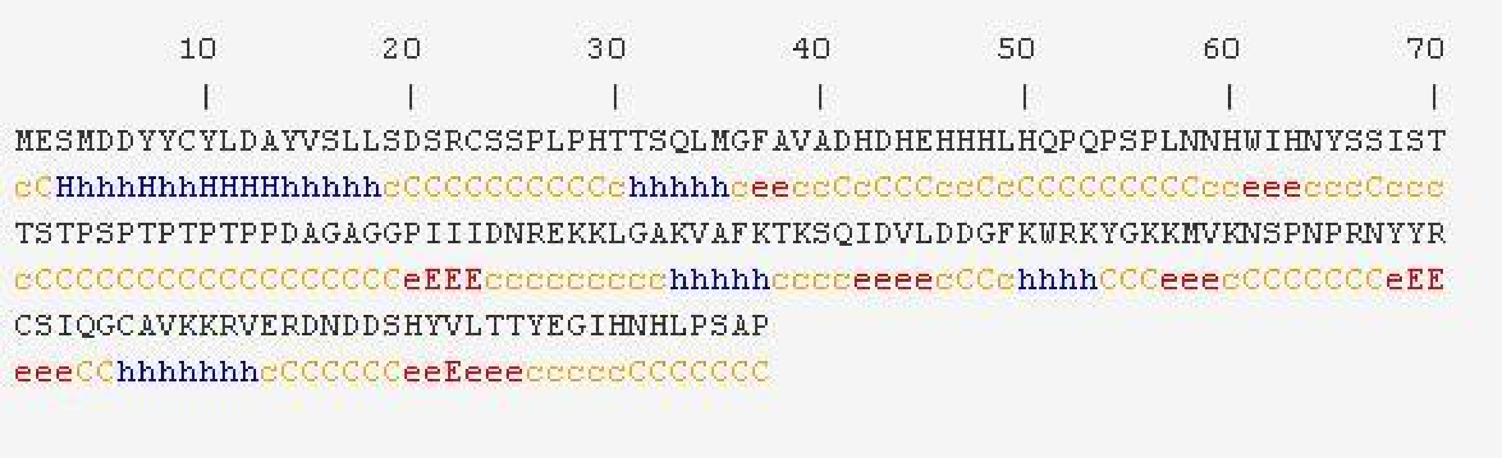

Supplementary Figure 3 Secondary structure of *BcWRKY1* protein

The secondary structural components of the protein demonstrated a ratio of the alpha helix, extended strand, and random coils on the website of (https://npsa-prabi.ibcp.fr/cgi-bin/npsa_automat.pl?page=/ NPSA/npsa_seccons.html)

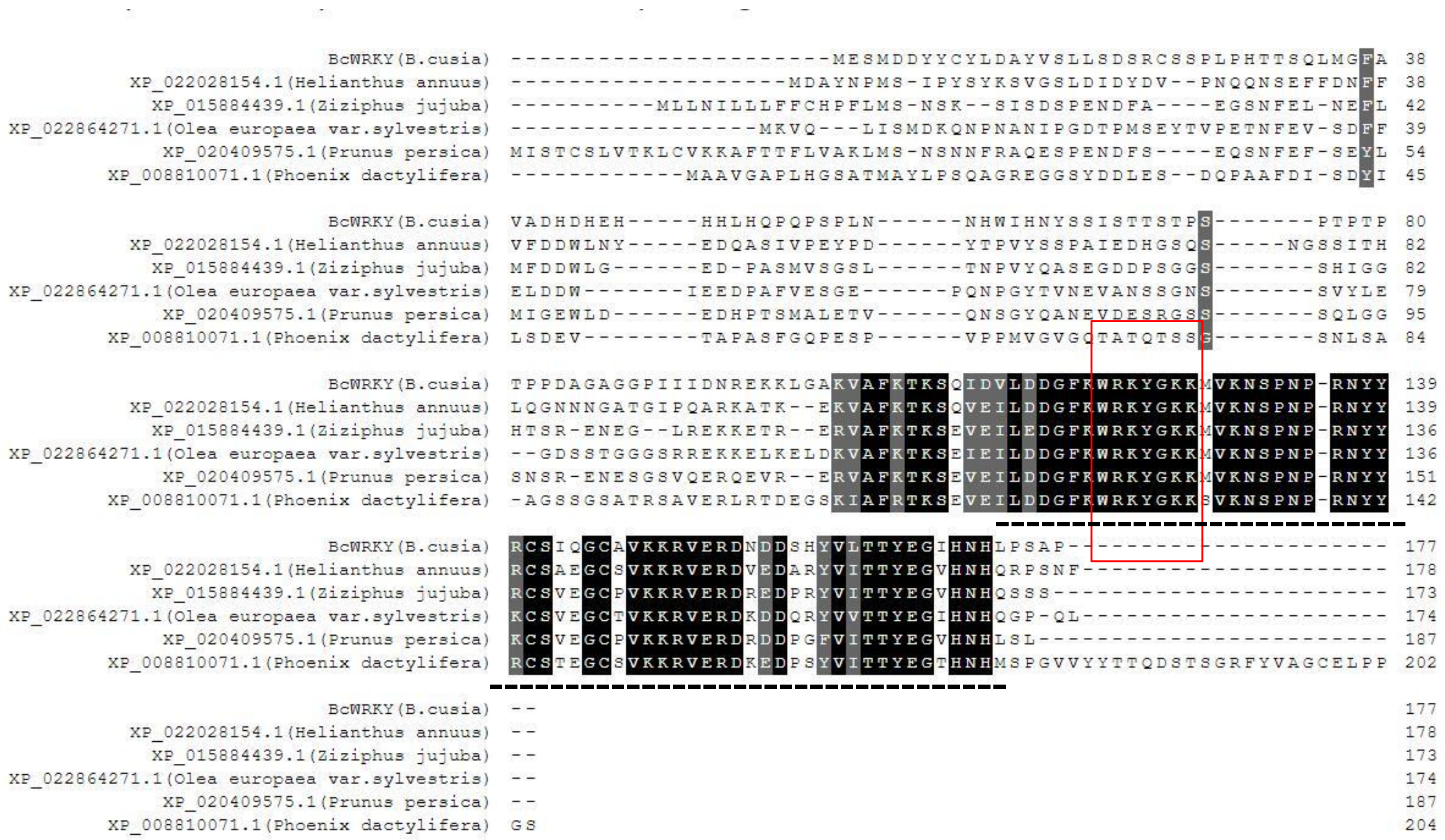

Supplementary Figure 4 Sequence homology analysis of *BcWRKY1* protein

The search for protein sequence similarities of *BcWRKY1* was conducted using BLAST algorithm at the National Center for Biotechnology Information (http://www.ncbi.nlm.nih.gov/blast). Conserved motif WRKYGKK in the frame is the characteristic sequence of WRKY transcription factor;The underlined part is the conservative domain of WRKY.

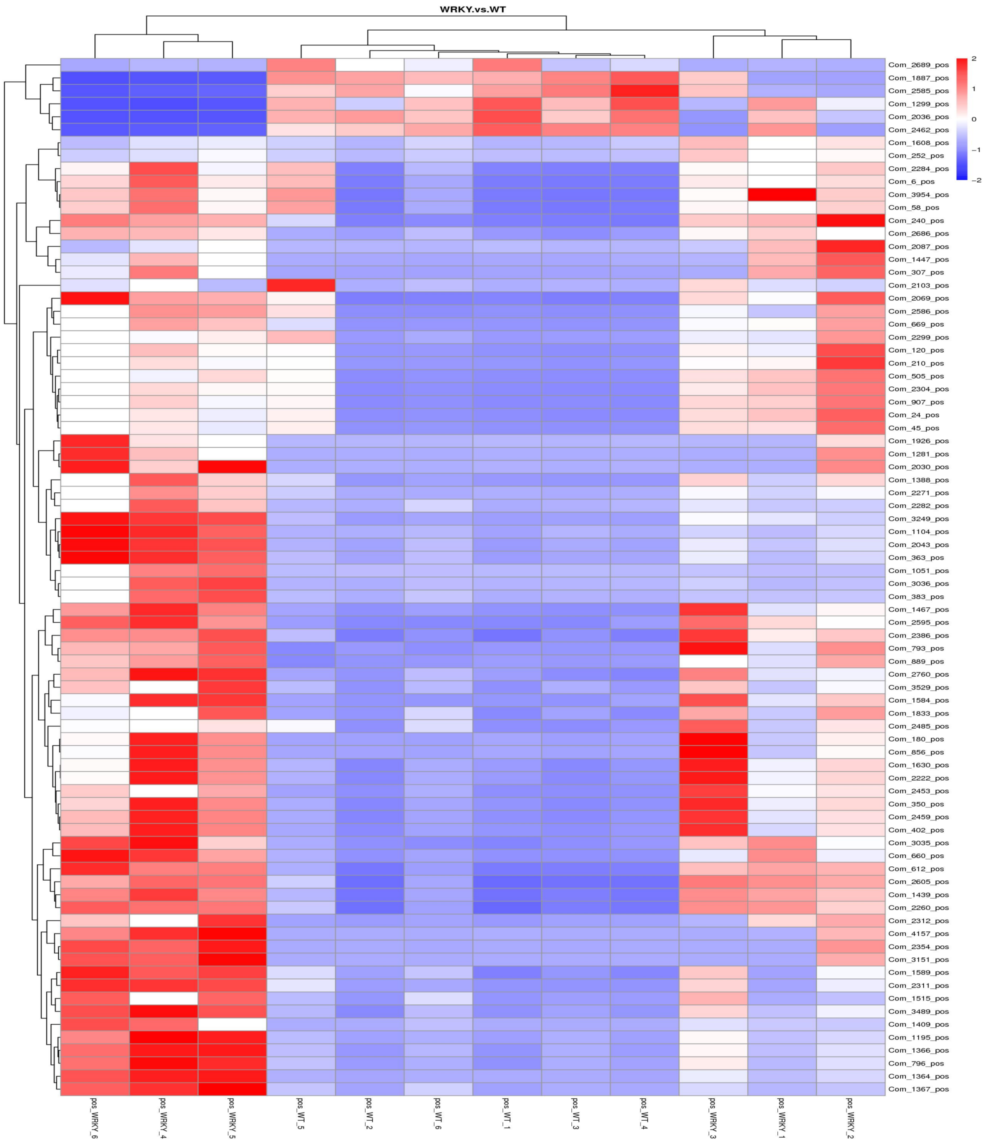

Supplementary Figure 5 Analysis of differential metabolites between wild type and over-expressed *BcWRKY1*

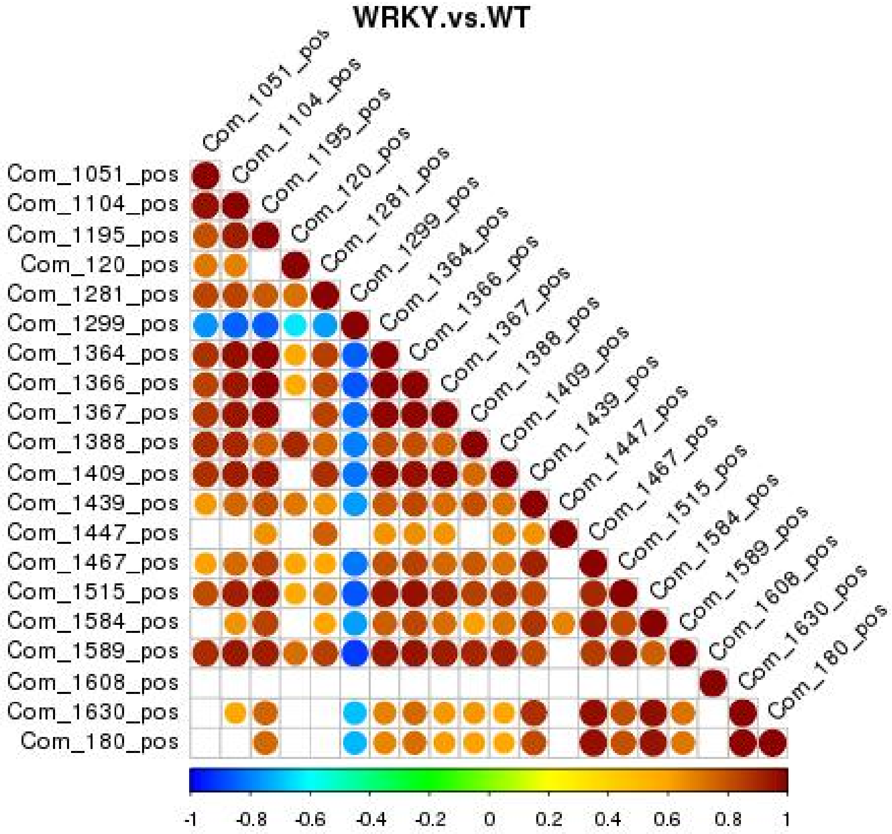

Supplementary Figure 6 Correlation analysis of differential metabolites

The correlation analysis of differential metabolites with cor.mtest in R. P value < 0.05.

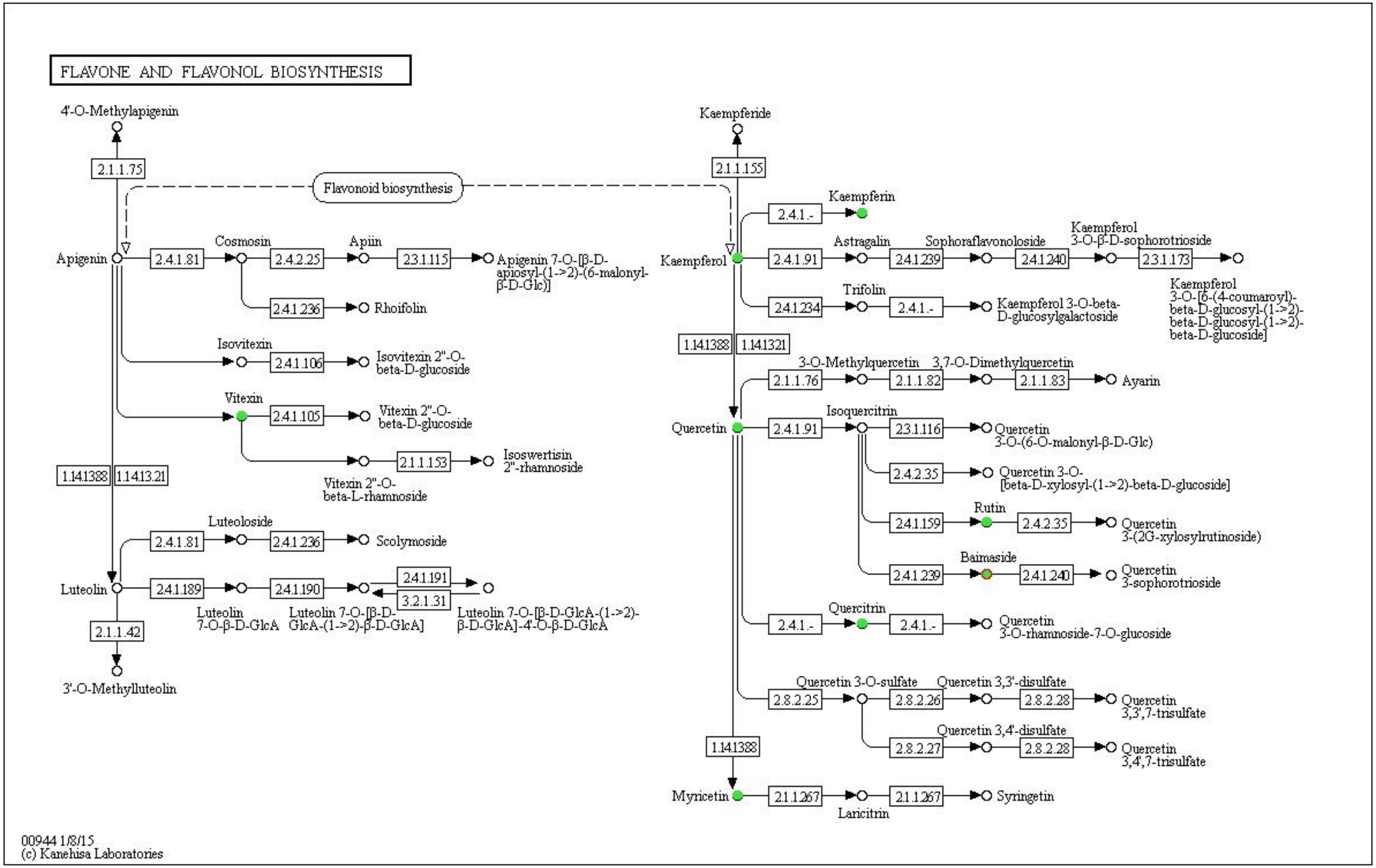

Supplementary Figure 7 Enrichment analysis of Flavone pathway and relative metabolites

The flavone metabolism pathway and relative metabolites in the pathway significantly influenced by the over-expression of *BcWRKY1*.

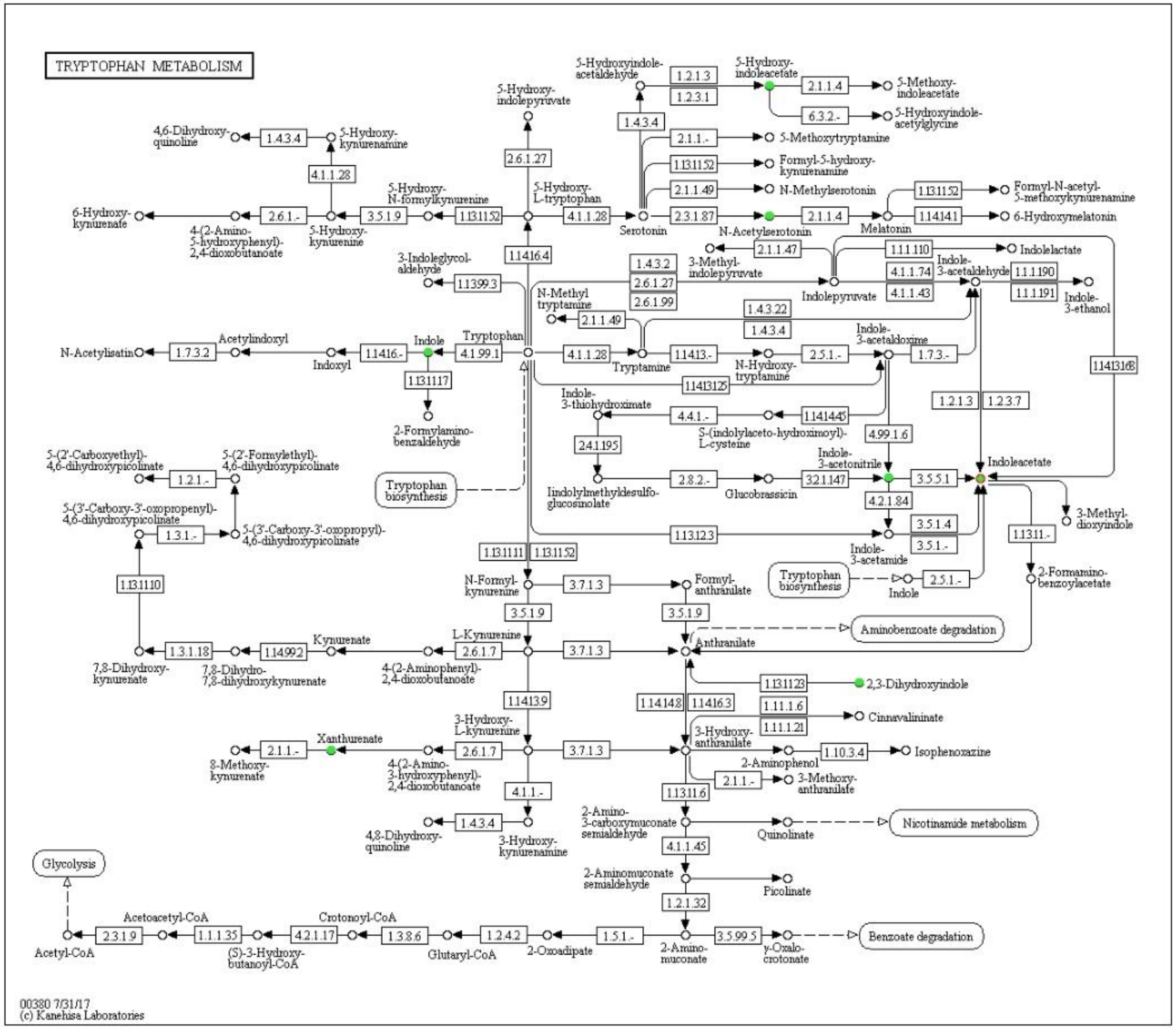

Supplementary Figure 8 Enrichment analysis of Tryptophan pathway and relative metabolites

The Tryptophan metabolism pathway and relative metabolites in the pathway significantly influenced by the over-expression of *BcWRKY1*.
