# Supplementary material for: Expression and Functional Study of BcWRKY1 in Baphicacanthus cusia (Nees) Bremek
Source: Front Plant Sci. 2022 Jul 1;13:919071. doi: 10.3389/fpls.2022.919071 (PMC9284225; doi:10.3389/fpls.2022.919071)
Supplement: Supplementary file 2 [file Table_1.docx]

**Supplementary Table 1** Composition and physicochemical characteristics comparisons of the deduced BcWRKY1 protein

| Composition and physicochemical characteristics comparisons of the deduced BcWRKY1 protein | | | |
| --- | --- | --- | --- |
| Number of amino acids (aa) | 177 | Formula | C_876_H_1338_N_250_O_269_S_8_ |
| Molecular weight  (kD) | 19.93 | Theoretical isoelectric point (pI) | 7.2 |
| Instability index | 43.21 | Grand average of hydrophobicity (GRAVY) | -0.818 |
| The total number of negatively charged residues (Asp + Glu) | 19 | The total number of positively charged residues (Arg + Lys) | 19 |
| Amino acid composition (%) | Ala (A) 5.1 Arg (R) 4.0 Asn (N) 5.1  Asp (D) 7.9 Cys (C) 2.3 Gln (Q) 2.8  Glu (E) 2.8 Gly (G) 5.1 His (H) 6.8  Ile (I) 4.5 Leu (L) 6.2 Lys (K) 6.8  Met (M) 2.3 Phe (F) 1.7 Pro (P) 9.0  Ser (S) 10.2 Thr (T) 6.2 Trp (W) 1.1  Tyr (Y) 5.6 Val (V) 4.5 | | |
