# Supplementary material for: Expression and Functional Study of BcWRKY1 in Baphicacanthus cusia (Nees) Bremek
Source: Front Plant Sci. 2022 Jul 1;13:919071. doi: 10.3389/fpls.2022.919071 (PMC9284225; doi:10.3389/fpls.2022.919071)
Supplement: Supplementary file 3 [file Table_2.docx]

**Supplementary Table 2** Nucleotide sequences of primers used in this study

| Primer names | Primer sequence |
| --- | --- |
| WRKY-F-XhoI | CCGCTCGAGATGGAGAGCATGGATGATTATTATT |
| WRKY-R-BamHI | CGGGATCCTTAAGGTGCAGAAGGTAGATGATTGC |
| WRKY-for-5941GFP-F | TTACAATTACCATGGGGCGCGCCATGGAGAGCATGGATGATTA |
| WRKY-for-5941GFP-R | GCCCTTGCTCACCATGGCGCGCCCAGGTGCAGAAGGTAGATGA |
| WRKY-R-for-BD-F | TGGCCATGGAGGCCGAATTCATGGAGAGCATGGATGATTA |
| WRKY-F-FOR-BD-R | CGACGGATCCCCGGGAATTCTTAAGGTGCAGAAGGTAGAT |
| Actin-F | GTTCTCGATGTTGTTCGTAAG |
| Actin-R | TGTAAGGCTCAACCACAGTAT |
| WRKY-qPCR-F | TGGTCAAGAACAGCCCTAATC |
| WRKY-qPCR-R | TGATTGTGGATGCCCTCATAG |
| W-box-F | ATGGCGCCATTTGACTAGCGTAGTTTGACCGCTCATGAACA |
| W-box-R | TGTTCATGAGCGGTCAAACTACGCTAGTCAAATGGCGCCAT |
| GST-WRKY-F | CGCGTGGATTCCCGGAATTCATGGAGAGCATGGATGATTA |
| GST-WRKY-R | TCGAGTCGACCCGGGAATTCTTAAGGTGCAGAAGGTAGAT |
| YUC1-F | CTCCGGTTTCTGGAGAGTAAAG |
| YUC1-R | GGAAGTATGGATCTGCGTTCTC |
| TAA1-F | GACATATGTTCGGTCGGGTATG |
| TAA1-R | GATGGTTCCGTCAGGGTTATTAG |
| CHS-F | TGACTGGAACTCCCTCTTCT |
| CHS-R | GCCCTCATCTTCTCTTCCTTTAG |
