# Supplementary material for: Expression and Functional Study of BcWRKY1 in Baphicacanthus cusia (Nees) Bremek
Source: Front Plant Sci. 2022 Jul 1;13:919071. doi: 10.3389/fpls.2022.919071 (PMC9284225; doi:10.3389/fpls.2022.919071)
Supplement: Supplementary file 4 [file Table_3.doc]

**Supplementary Table 3** Differential metabolites between wild type and *BcWRKY1-OX1* transgenic *Arabidopsis thaliana*

| Group | Total number of compounds identified | Total number of metabolites with significant differences | Total number of metabolites significantly up-regulated | Total number of metabolites significantly down-regulated |
| --- | --- | --- | --- | --- |
| BcWRKY.vs.WT | 2131 | 80 | 74 | 6 |
