# Supplementary material for: Expression and Functional Study of BcWRKY1 in Baphicacanthus cusia (Nees) Bremek
Source: Front Plant Sci. 2022 Jul 1;13:919071. doi: 10.3389/fpls.2022.919071 (PMC9284225; doi:10.3389/fpls.2022.919071)
Supplement: Supplementary file 5 [file Table_4.doc]

**Supplementary Table 4** KEGG Pathway enrichment analysis in wild type and *BcWRKY1-OX1* transgenic *Arabidopsis thaliana*.

| ID of  KEGG Pathway | KEGG Pathway  Name | P-value | Corrected P- value | Numbers of differential metabolites associated with the pathway | Numbers of background metabolites associated with the pathway | Numbers of differential metabolites of kegg notes | Numbers of background metabolites of kegg notes | List of Enriched metabolites |
| --- | --- | --- | --- | --- | --- | --- | --- | --- |
| [map00941](../../../../E:/%25E5%258D%258E%25E5%25A4%25A7-2018-04-30/%25E6%25AF%2595%25E4%25B8%259A%25E7%259B%25B8%25E5%2585%25B3%25E6%259D%2590%25E6%2596%2599/%25E4%25B8%258A%25E4%25BA%25A4%25E6%25AF%2595%25E4%25B8%259A%25E8%25AE%25BA%25E6%2596%2587-2019-02-08/P101SC18123438-01-B1-23.result/P101SC18123438-01-B1-23.result/4.MetKeggAnalysis/WRKY.vs.WT/WRKY.vs.WT_pos_KEGG_map/src/00941.html) | Flavonoid biosynthesis | 0.0419481949844043 | 0.309274423919253 | 2 | 9 | 7 | 178 | Com_24_pos Com_3954_pos |
| [map00940](../../../../E:/%25E5%258D%258E%25E5%25A4%25A7-2018-04-30/%25E6%25AF%2595%25E4%25B8%259A%25E7%259B%25B8%25E5%2585%25B3%25E6%259D%2590%25E6%2596%2599/%25E4%25B8%258A%25E4%25BA%25A4%25E6%25AF%2595%25E4%25B8%259A%25E8%25AE%25BA%25E6%2596%2587-2019-02-08/P101SC18123438-01-B1-23.result/P101SC18123438-01-B1-23.result/4.MetKeggAnalysis/WRKY.vs.WT/WRKY.vs.WT_pos_KEGG_map/src/00940.html) | Phenylpropanoid biosynthesis | 0.0725587287897821 | 0.309274423919253 | 2 | 12 | 7 | 178 | Com_3954_pos Com_6_pos |
| [map00945](../../../../E:/%25E5%258D%258E%25E5%25A4%25A7-2018-04-30/%25E6%25AF%2595%25E4%25B8%259A%25E7%259B%25B8%25E5%2585%25B3%25E6%259D%2590%25E6%2596%2599/%25E4%25B8%258A%25E4%25BA%25A4%25E6%25AF%2595%25E4%25B8%259A%25E8%25AE%25BA%25E6%2596%2587-2019-02-08/P101SC18123438-01-B1-23.result/P101SC18123438-01-B1-23.result/4.MetKeggAnalysis/WRKY.vs.WT/WRKY.vs.WT_pos_KEGG_map/src/00945.html) | Stilbenoid, diarylheptanoid and gingerol biosynthesis | 0.0773186059798134 | 0.309274423919253 | 1 | 2 | 7 | 178 | Com_3954_pos |
| [map04075](../../../../E:/%25E5%258D%258E%25E5%25A4%25A7-2018-04-30/%25E6%25AF%2595%25E4%25B8%259A%25E7%259B%25B8%25E5%2585%25B3%25E6%259D%2590%25E6%2596%2599/%25E4%25B8%258A%25E4%25BA%25A4%25E6%25AF%2595%25E4%25B8%259A%25E8%25AE%25BA%25E6%2596%2587-2019-02-08/P101SC18123438-01-B1-23.result/P101SC18123438-01-B1-23.result/4.MetKeggAnalysis/WRKY.vs.WT/WRKY.vs.WT_pos_KEGG_map/src/04075.html) | Plant hormone signal transduction | 0.149455514966846 | 0.431684118823667 | 1 | 4 | 7 | 178 | Com_252_pos |
| [map00380](../../../../E:/%25E5%258D%258E%25E5%25A4%25A7-2018-04-30/%25E6%25AF%2595%25E4%25B8%259A%25E7%259B%25B8%25E5%2585%25B3%25E6%259D%2590%25E6%2596%2599/%25E4%25B8%258A%25E4%25BA%25A4%25E6%25AF%2595%25E4%25B8%259A%25E8%25AE%25BA%25E6%2596%2587-2019-02-08/P101SC18123438-01-B1-23.result/P101SC18123438-01-B1-23.result/4.MetKeggAnalysis/WRKY.vs.WT/WRKY.vs.WT_pos_KEGG_map/src/00380.html) | Tryptophan metabolism | 0.248581751246402 | 0.431684118823667 | 1 | 7 | 7 | 178 | Com_252_pos |
| [map00944](../../../../E:/%25E5%258D%258E%25E5%25A4%25A7-2018-04-30/%25E6%25AF%2595%25E4%25B8%259A%25E7%259B%25B8%25E5%2585%25B3%25E6%259D%2590%25E6%2596%2599/%25E4%25B8%258A%25E4%25BA%25A4%25E6%25AF%2595%25E4%25B8%259A%25E8%25AE%25BA%25E6%2596%2587-2019-02-08/P101SC18123438-01-B1-23.result/P101SC18123438-01-B1-23.result/4.MetKeggAnalysis/WRKY.vs.WT/WRKY.vs.WT_pos_KEGG_map/src/00944.html) | Flavone and flavonol biosynthesis | 0.279341562598888 | 0.431684118823667 | 1 | 8 | 7 | 178 | Com_307_pos |
| [map04976](../../../../E:/%25E5%258D%258E%25E5%25A4%25A7-2018-04-30/%25E6%25AF%2595%25E4%25B8%259A%25E7%259B%25B8%25E5%2585%25B3%25E6%259D%2590%25E6%2596%2599/%25E4%25B8%258A%25E4%25BA%25A4%25E6%25AF%2595%25E4%25B8%259A%25E8%25AE%25BA%25E6%2596%2587-2019-02-08/P101SC18123438-01-B1-23.result/P101SC18123438-01-B1-23.result/4.MetKeggAnalysis/WRKY.vs.WT/WRKY.vs.WT_pos_KEGG_map/src/04976.html) | Bile secretion | 0.337636383640315 | 0.431684118823667 | 1 | 10 | 7 | 178 | Com_1584_pos |
| [map01061](../../../../E:/%25E5%258D%258E%25E5%25A4%25A7-2018-04-30/%25E6%25AF%2595%25E4%25B8%259A%25E7%259B%25B8%25E5%2585%25B3%25E6%259D%2590%25E6%2596%2599/%25E4%25B8%258A%25E4%25BA%25A4%25E6%25AF%2595%25E4%25B8%259A%25E8%25AE%25BA%25E6%2596%2587-2019-02-08/P101SC18123438-01-B1-23.result/P101SC18123438-01-B1-23.result/4.MetKeggAnalysis/WRKY.vs.WT/WRKY.vs.WT_pos_KEGG_map/src/01061.html) | Biosynthesis of phenylpropanoids | 0.365234867655302 | 0.431684118823667 | 1 | 11 | 7 | 178 | Com_6_pos |
| [map01070](../../../../E:/%25E5%258D%258E%25E5%25A4%25A7-2018-04-30/%25E6%25AF%2595%25E4%25B8%259A%25E7%259B%25B8%25E5%2585%25B3%25E6%259D%2590%25E6%2596%2599/%25E4%25B8%258A%25E4%25BA%25A4%25E6%25AF%2595%25E4%25B8%259A%25E8%25AE%25BA%25E6%2596%2587-2019-02-08/P101SC18123438-01-B1-23.result/P101SC18123438-01-B1-23.result/4.MetKeggAnalysis/WRKY.vs.WT/WRKY.vs.WT_pos_KEGG_map/src/01070.html) | Biosynthesis of plant hormones | 0.391841789370349 | 0.431684118823667 | 1 | 12 | 7 | 178 | Com_252_pos |
| [map01110](../../../../E:/%25E5%258D%258E%25E5%25A4%25A7-2018-04-30/%25E6%25AF%2595%25E4%25B8%259A%25E7%259B%25B8%25E5%2585%25B3%25E6%259D%2590%25E6%2596%2599/%25E4%25B8%258A%25E4%25BA%25A4%25E6%25AF%2595%25E4%25B8%259A%25E8%25AE%25BA%25E6%2596%2587-2019-02-08/P101SC18123438-01-B1-23.result/P101SC18123438-01-B1-23.result/4.MetKeggAnalysis/WRKY.vs.WT/WRKY.vs.WT_pos_KEGG_map/src/01110.html) | Biosynthesis of secondary metabolites | 0.395710442255028 | 0.431684118823667 | 3 | 49 | 7 | 178 | Com_24_pos Com_3954_pos Com_6_pos |
|  |  |  |  |  |  |  |  |  |
